# Emerging and re-emerging infectious diseases in Iran

**Published:** 2017-06

**Authors:** Najmeh Parhizgari, Mohammad Mehdi Gouya, Ehsan Mostafavi

**Affiliations:** 1 Department of Virology, School of Public Health, Tehran University of Medical Sciences, Tehran, Iran; 2 Department of Epidemiology and Biostatistics, Research Centre for Emerging and Reemerging Infectious Diseases, Pasteur Institute of Iran, Tehran, Iran; 3 The Center for Communicable Diseases Control, Department for Health Affairs, Ministry of Health and Medical Education, Tehran, Iran; 4 National Reference Laboratory for Plague, Tularemia and Q Fever, Research Centre for Emerging and Reemerging Infectious Diseases, Pasteur Institute of Iran, Akanlu, Kabudar Ahang, Hamadan, Iran

**Keywords:** Epidemiology, Public health, Infectious diseases, Plague, Tuberculosis, Dengue

## Abstract

Despite development of preventive and controlling strategies regarding infectious diseases, they are still considered as one of the most significant leading causes of morbidity and mortality, worldwide. Changes in humans’ demographics and behaviors, microbial and ecological alterations, agricultural development, international travels and susceptibility to infectious diseases have resulted in increased reports of emerging infectious diseases (EIDs) and reemerging infectious diseases (RIDs) in various geographical areas.

Because of the various types of geographic properties in Iran, substantial climatic variability, as well as unstable political situations and poor public health conditions in some of neighboring countries, EIDs and RIDs are serious public health problems; among them, zoonotic and drug resistant diseases are the most significant.

Hence, this review provides an overview of the significant bacterial, viral and fungal EIDs and RIDs in Iran regarding their epidemiological aspects.

## INTRODUCTION

Over the past century, even thought there was considerable development regarding prevention, control and elimination of some of the infectious diseases through proper use of hygiene and sanitation practices in addition to development of antibiotics and vaccination, some infectious diseases remained as the leading causes of morbidity and mortality worldwide. Furthermore, amongst the challenges in controlling these infectious diseases, emerging infectious diseases (EIDs) and reemerging infectious diseases (RIDs) could be pointed out (1).

EIDs are diseases that occur for the first time in the world, a defined region or a given population, or diseases that already existed but would emerge with a different pattern of virulence or resistance to current drugs (2–4). RIDs are infectious diseases which have been eradicated or reduced in a way that they did not cause any serious public health problem; however, once again, they show upward trends in incidence in a specific population or region (2, 3). Many EIDs and RIDs are zoonotic, can be transmitted from animals to human hosts. As the diseases in reservoirs of zoonotic EIDs or RIDs can be asymptomatic, the new reports of these diseases among human can reflect the infection of their reservoir hosts (3, 5, 6).

Changes in humans’ demographics and behaviors, microbial and ecological alterations, agricultural development, breakdown of public health measures, international commerce, war and famine and susceptibility to infectious diseases have resulted in increased reports of EIDs and RIDs in various geographical areas (2–4). In recent years, increased international travels have also led to spread of EIDs and RIDs; therefore, they were not easily controlled (7–9).

Like many other countries, EIDs and RIDs are considered a significant public health problem in Iran which is the 18^th^ largest country in the world, geographically located in the Middle East. With over 77 million inhabitants, Iran is the world’s 17^th^ most populous country and shares land borders with Pakistan, Turkmenistan, Armenia, Azerbaijan, Turkey, Iraq, Afghanistan and maritime borders with Saudi Arabia, United Arab Emirates Kuwait, Kazakhstan and Russia. Each of these countries has some endemic infectious diseases that can be a threat to their neighboring countries, including Iran (10, 11). Unstable political conditions and wars in some of these countries have resulted in migrations and dislocations which are important factors in the spread of such endemic infectious diseases (6). Legal and illegal immigrants and refugees from these countries and international travels can be the source of some of EIDs or RIDs in Iran (12).

Therefore, this paper reviews the status of significant EIDs and RIDs, based on the existing reports. The review is divided into viral, bacterial, parasitic and fungal infectious diseases and can serve as a practical summary to be used by physicians, health care workers and researchers who would encounter these diseases in Iran.

### Viral EIDs and RIDs

#### Crimean Congo Hemorrhagic Fever.

Crimean Congo Hemorrhagic Fever (CCHF) is the most significant tick-borne viral human infection which is reported sporadically across a vast geographic area with endemic patterns in Asia, Africa, Eastern Europe and the Middle East (13).

The first infection by CCHF virus (CCHFV) in Iran was reported in the early 1970s, when CCHFV antibodies were detected in livestock and human serum samples (14–16). Between 1974 and 1975, sero-conversion was founded among humans (13%), sheep (18%) and cattle (38%) in the northern parts of the country (17). In 1978, CCHFV was isolated from ticks in the North-east of Iran (18). The first confirmed human case of CCHF was reported in 1999 in Chaharmahal and Bakhtiari Province, central region of Iran, and also redounded as the first case of nosocomial infection in the country (19). The number of CCHF-endemic provinces has substantially increased in recent years (20–22) and it is reported from almost all parts of the country (23, 24).

To date, several CCHF outbreaks have occurred in Iran (25, 26). The majority of confirmed human cases in Iran have been butchers, slaughterhouse workers and farmers, who mainly deal with infected livestock (27–31). Most of the infected cases have occurred in south-eastern Iran, Sistan and Baluchistan province, close to the borders of Afghanistan and Pakistan. The large number of confirmed cases in Sistan and Baluchistan province can be the result of a higher awareness among the physicians in respect with the infection in this region and the hyper-endemicity of CCHF in Afghanistan and Pakistan. An additional factor is the large number of afghan refugees and immigrants in this province in a way that some of them come to Iran just for CCHF treatment (24). Analyzing the CCHFV genome has led to the identification of more than five circulating CCHFV genomic variants in Iran (25, 29, 32–34).

Since 1975, the surveys on livestock have revealed 3.8 to 100% infection rate in different parts of the country (35–37). In 2007, ostriches were first identified as the probable host transmitting the disease to humans in Iran (38). The virus was also isolated among 4.3 to 28% of the ticks studied in different areas (36, 39–42). It was shown that an increase in the temperature and also a decrease in rainfall have enhanced the activity of ticks and raised the number of reported CCHF cases (43).

To control the disease, the import of livestock into the country, especially via the eastern borders, should be monitored and the populations of livestock and ticks in high-risk areas and endemic regions should be systematically surveyed for further interventions.

#### West Nile Fever.

West Nile Virus (WNV) is a mosquito-borne virus isolated from birds, horses, mosquitoes and human patients (44). Birds are the primary natural reservoirs of the virus and the infection is mainly found in wetland ecosystems (45). WNV is the most widespread *Flavivirus*, distributed in Africa, America, Australia, South of Europe, West of Asia and the Middle East and has been found in different neighboring countries of Iran (46, 47).

Serological investigations in the 1970s showed the presence of WNV antibody in human populations in several provinces in Iran (15, 48). A WNV survey from 2004 to 2007 on migratory wild birds of the wetlands in different parts of Iran has revealed the presence of 15% of WNV antibody among them (49). In a cross-sectional study performed on blood donors in Tehran in 2005, five percent of the donors were seropositive for WNV (50). Between 2008 and 2009, 1.2% of all patients with fever and loss of consciousness in Isfahan province, in the central part of the country, were WNV positive using RT–PCR (51). In a study in 2010–2011 in the northern and central provinces of Iran, 1.3% of the general population and 2.8% of the horses were seropositive for WNV (52). In 2009, a large-scale sero-survey of the equine population in various regions indicated that 23.7% of the horses were positive for WNV antibodies (53). Moreover, in southwestern Iran, WNV antibody was reported among 70% of the horses between 2011 to 2012 (54).

As the WNV antibody is shown in humans, wild birds and horses in different studies in recent years, it seems that the country is facing with this infectious disease in most parts and the surveillance system should be more active in case finding, reporting and treatment.

#### Dengue Fever.

Dengue Fever (DF) is one of the most problematic arboviral diseases in human populations. The transmission of dengue virus (DV) is geographically extended over the recent decades in a way that all dengue virus serotypes (DV1-4) are now circulating in Asia, Africa and the Americas, as the result of global warming and changes in social behavior, urbanization and globalization and an increase in international travels (55, 56).

*Aedes aegypti*, which is the most significant vector of DV, has not been reported in Iran. In 2008, the first case of dengue fever was reported in Iran in a patient that had previously travelled to Malaysia (57). In a study among 300 Iranian patients tested negative for CCHFV between 2000 and 2012, 5% were serologically or molecularly positive for DF. In this study, most of the positive cases had travelled to endemic areas including Malaysia, India and Thailand (58). In another study in Sistan and Baluchistan province in southeastern Iran, approximately 6% of all blood donors were asymptomatically seropositive for DV (59).

Because of the important role of travelers in the import of DF cases in Iran, precautionary measures such as wearing insect repellent and applying protective cover should be recommended to travelers to endemic areas to avoid mosquito bites (58); more entomological studies is recommended in order to clarify the situation of potential vectors in Iran.

#### HIV/AIDS.

Acquired Immune Deficiency Syndrome (AIDS), a globally distributed infectious disease, was first identified in hemophilia patients in 1986 in Iran (60). In 2013, it was estimated that there were almost 70,000 HIV-infected people in the country.

Iran is among countries with concentrated epidemic level of HIV; the infection remains low in the general population (<1%) (61), but certain groups such as injecting drug users (IDUs) (10.7%), prison inmates (13.2%), female sex workers (<5%) and homeless people (<4%) were shown to be at greater risk of the infection (62–68). HIV transmission by injecting drug use has affected a lot of people in Iran (69), however recent studies have shown that the transmission pattern is changing from drug injection to sexual practice (70). Harm reduction programs have been successful in reducing the transmission of HIV among high-risk groups in Iran (71).

Phylogenetic analysis of HIV indicates that HIV-1 subtype B and A are the frequent subtype among hemophilia patients and injecting drug users, respectively; suggesting that HIV infection in Iran has at least two origins (72).

Additionally, cultural changes and increased risky behavior among young people in this country have increased the vulnerability of the country to HIV/AIDS (73–75).

As it is demonstrated that increasing the knowledge and awareness regarding HIV/AIDS transmission and prevention is one of the most crucial preventive methods among different groups (76), increasing the training programs and improving the quality of harm reduction programs are highly recommended to control the disease in Iran.

#### Hepatitis C.

Hepatitis C virus (HCV) has affected about 175 million people worldwide and is considered as one of the leading cause of liver transplantation (77, 78). In Iran, the virus is introduced as an emerging viral infection amongst high risk populations like injecting drug users as this group has shown a higher prevalence of HCV in recent years (79).

A study conducted in 1994 on healthy blood donors, revealed 0.25% of seroconversion for HCV infection for the first time in Iran (79). HCV infection prevalence has a low rate in general population in Iran compared to the adjacent countries of Pakistan, Turkey and Iraq (80–82). The infection amongst blood donors is 0.1 to 0.5% in different cities of the country (83–86).

Different dialysis centers have had diverse frequency of HCV infection ranging from 5 to 23.9% (87, 88). The main route of HCV transmission among hemophilia and thalassemia patients is through blood products (89–93). In years 1999 and 2000, 0.59% of HCV antibody positive cases were confirmed in multi-transfused children with β-thalassemia in Shiraz blood bank (94). In 2005, a multicenter study pointed out that 19.3% of thalassemia patients suffered from HCV infection (91). In 2007, the infection rate varied between 15.7% and 63.8% (95). In recent years, other serological studies have shown that 15 to 91% of all patients with hemophilia have antibodies against HCV (96–99). This evidence emphasizes the importance of screening of hemophilic patients for HCV infection.

The HCV genome pattern has changed during recent years in Iran and it seems that such a change can be due to cross-border travels between Iran, Pakistan and Iraq (82).

To decrease the trend of infection, regular surveys and interventions should be done, focusing on high-risk groups such as IDUs, those who receive blood products and health care workers with occupational exposure (99).

#### Occult hepatitis B virus infection.

Occult hepatitis B virus infection (OBI) is an emerging type of HBV infection when HBV DNA is detectable among HBsAg negative infected patients (100). Attention to OBI has increased due to its potential role in accelerating the progression of liver fibrosis and cirrhosis, ultimately leading to hepatocellular carcinoma (HCC); It is transmitted via blood transfusion and transplantation (100).

Introduction of the occult infection was the consequence of improvement of HBV DNA detection and introduction of more sensitive methods which were not available earlier than 1985 (101). OBI seems to be highly prevalent in Asia (102). The first evidence of OBI in Iran refers to 2001–2002 when 22% of chronic liver patients were revealed to be positive for HBV genome tests (103). It has been detected amongst 30% of high risk groups as well as hemodialysis patients and is considered common in HIV patients (104–106).

As OBI has been found among a large proportion of HBcAb positive healthy blood donors, thus the blood of these people should be screened in blood transfusion centers to prevent HBV transmission (84, 107–109).

#### Human T-cell leukemia.

The Human T-cell leukemia virus (HTLV), the first known human oncogenic retrovirus, is the causative agent of HTLV-I-associated myelopathy/tropical spastic paraparesis (HAM/TSP) and adult T-cell leukemia (ATL) (110, 111). It is present all over the world with clusters of high endemicity in Japan, sub-Saharan Africa, South America, the Caribbean area and some foci in the Middle East (112). The HTLV-1 was first reported in the Middle East in 1983 and the first clinical report matching to ATL in Iran was from Mashhad, in northeastern Iran, in 1986 (113, 114). There were continuous reports of HTLV-1 infection among blood donors and various patients from Mashhad from 1991 to 1993 (115–122). A molecular study showed that the virus clustered in the cosmopolitan subtype (123). In 1993, a blood donor-based study showed a low rate (0–0.5%) of infection in other parts of the country. HTLV-1 then showed a slight increase (3%) among Mashhadi blood donors until 1995 (122). Afterwards, the rate of infection decreased during the next three years to 2%, 0.77% and 0.45%, respectively (124, 125). A study in 2011 revealed 2.12% HTLV-1 positive cases among the general population in Mashhad suggesting a reemerging status of the infection in this city and its neighboring city, Neyshabour (126–128).

Frequent blood recipients and hemodialysis patients remain the major high-risk groups for HTLV-1 infection (1–7%) around the country (129–131). In addition to blood transfusion, breast-feeding and sexual transmission, history of surgical procedures, hospitalization and traditional cupping were introduced as other main risk factors for HTLV-1 transmission in Iran (126, 132).

Iran has the second rank for HTLV-1 prevalence after Japan (133). Proper screening measures can be included in blood transfusion centers in the country. Educational programs, in addition to highly sensitive screening tests, seem necessary in health centers to reduce the transmission of the infection (134).

#### H1N1 Flu.

A highly infectious type of Influenza virus, H1N1, was emerged in April 2009 in Mexico. The virus rapidly spread through USA and Canada and caused a pandemic. WHO announced a world threat of the disease and asked the countries to screen the infection in all suspected patients (135, 136).

Geographic distribution of the reported cases in Iran showed that the highest reported cases belonged to the central and eastern provinces. Till March 2010, 3672 cases of H1N1 were confirmed and 147 patients (4.0%) died in Iran accordingly (137, 138). The results of a surveillance system in Kurdistan, a west frontier province of Iran, during 2009–2010 showed 157 positive H1N1 cases among 1059 suspected patients. It seems that the virus had spread to Kurdistan after entering to Iran by traveling (139). A survey in Shiraz airport, south of Iran, on hajj pilgrims who returned from Mecca in 2009 revealed 1.6% of the infection with swine influenza (140).

Screening of travelers in entering borders is suggested for early detection and inhibition of the pathogen (140). On the other side, the surveillance system should be ready to screen the high risk groups and suspected cases to rapidly detect and control the disease.

#### Middle East Respiratory Syndrome.

The Middle East Respiratory Syndrome-Corona virus (MERS-CoV), a newly recognized corona virus, emerged in Qatar and Saudi Arabia with a high fatality rate (50%) in 2012 (141).

The United Arab Emirates, Jordan, Oman, Kuwait and Egypt have all reported positive cases with recorded deaths (142). Several European and Asian countries such as the United Kingdom, France, Germany and Malaysia have also declared confirmed MERS-CoV patients (143, 144).

Dromedary camels (*Camelus dromedarius*) are considered as the natural reservoirs for MERS-CoV (145).

For the first time, the MERS-CoV RNA was confirmed in a woman in May 2014, who was admitted to a hospital in Kerman province in southern Iran. She died of progressive respiratory failure. The next three cases were the patient’s sister, nurse and physician who had close contact with her. The origin of the virus was contact with a woman who had an influenza-like illness and had traveled to Saudi Arabia two weeks earlier. The fifth patient was a 67-year old woman who was admitted to the hospital and died of MERS-CoV (146).

By the end of 2014, eight MERS-CoV cases were confirmed among camels, illegally imported from Pakistan into Sistan and Baluchistan province in south-eastern Iran (147). Nosocomial infection is an important mode of transmission for respiratory viruses, thus special infection control measures should be performed to prevent the possible spread of MERS-CoV within health care centers (141, 148). Considering that camels are common livestock in some parts of the country, awareness among their owners regarding MERS-CoV is important to prevent the possible spread of the disease (149).

#### Hantavirus Infection.

Hantavirus, an agent that is transmitted from rodents, has emerged with significant morbidity and mortality in humans as Hemorrhagic Fever with Renal Syndrome (HFRS) in Europe and Asia, and as Hantavirus Pulmonary Syndrome (HPS) in USA (150, 151). The disease has been reported in Turkey, a neighboring country of Iran (152, 153).

For the first time, in 2013, the existence of infection amongst individuals was shown by serological and molecular tests among the street cleaners in the central region of the country. In this study, the prevalence of rodents in the work place was a risk factor for being positive (154).

As there is no other report of Hantavirus infection in Iran, physicians and health care workers do not have enough information about the disease. Hence, it is recommended to conduct seroepidemiological surveys in humans and rodents in various geographical regions of Iran in order to identify the true situation and risk factors for acquiring a Hantavirus infection.

### Bacterial EIDs and RIDs

#### Q Fever.

*Coxiella burnetii* is an obligate intracellular bacterium, developing spore-like forms that cause Q fever. It is a public health problem and an occupational zoonotic disease, globally (155).

The first human Q fever infection in Iran was reported in Abadan, southwestern Iran, in 1952 (161). The disease was reported in humans and domestic animals from most parts of the country during the period between 1954 and 1959 (156, 157). The next Q fever case was reported 50 years later in 2009, as an RID in Bardsir, southern Iran (157). Butchers and other slaughterhouse workers are considered high-risk groups in west and southeast of the country with 68% and 38% seropositivity, respectively (158–160).

Recent research has introduced goats as the major reservoir of the bacterium in Iran, having the highest seroprevalence (66–69%) among livestock (161). Sheep and bovine Q fever seropositivity ranges from 13 to 30% in different provinces in the central and border regions (158, 162). In addition, it is a major cause of abortion in animals with the *C. burnetii* genome being found in aborted fetuses in ten provinces (163). The contamination of raw milk with *C. burnetii* has been identified in different regions of Iran (164–166). Ticks would also play an important role in the transmission of Q fever, acting as the reserves and vectors of *C. burnetii*. A study on ticks collected from goats and sheep have shown *Hyalomma* and *Rhipicephalus* as the main contaminated vectors in Iran (167).

Because of the complications of differential diagnosis of Q fever with other infectious diseases, and limited educational plans for physicians and specialists, Q fever attracts relatively little attention from public health workers in Iran. Increasing the knowledge of the physicians and veterinarians regarding the diseases is important. Q-fever should be considered as a differential diagnosis in case of influenza-like symptoms, pneumonia, hepatitis or endocarditis.

#### Plague.

Plague is caused by *Yersinia pestis.* Plague pandemics have killed millions of humans in Africa, Europe, Asia and America (168, 169).

Most of Iran’s neighbors have reported the disease during the last centuries; a recent outbreak of plague was reported in Afghanistan in 2007 (170). Iraq experienced multiple epidemics of plague in the eighth, eighteenth, nineteenth and twentieth centuries (171–173). Kazakhstan and Azerbaijan, to the north of the country, are important foci for the disease and are considered major potential sources of the infection (174, 175).

In Iran, plague is an ancient disease that has been recorded since the sixth century and has caused a vast range of mortality (176–178). Kurdistan and Kermanshah provinces in the west, Khorasan province in the east, East Azerbaijan, Zanjan and Ardabil provinces in the northwest and Bushehr in the south were profoundly affected by plague in the 19th century (176, 179–181).

In the 20th century, plague vaccination programs were conducted and resulted in the control of the disease, whilst it still remains endemic in Kurdistan province in western Iran (169, 182). *Y. pestis* has been isolated from wild rodents, as the main reservoirs, in Kurdistan province in various surveys (174, 183). A recent study in 2011–2012 showed persistence of plague infection in dogs and rodent population in Kurdistan (174, 184).

Plague should be considered as a potential RID in Iran and monitoring the disease, mainly in the western part of the country is important.

#### Tularemia.

*Francisella tularensis*, the causative agent of Tularemia, is a zoonotic pathogen (185) which has been only detected in the Northern Hemisphere, including Asia, Europe and the U.S. (186). Farmers, veterinarians, hunters, butchers, cooks and laboratory staff have the most risk of the infection (187–189). There are human and animal tularemia reports from countries neighboring Iran including Azerbaijan, Afghanistan, Armenia and Turkey (190–192).

In Iran, the first serological evidence of tularemia infection in animals dates back to 1973, when tularemia antibodies were detected in sheep, cows and porcupine in the northwest and southeast of the country (193). The clinical form of tularemia was reported in a patient in Marivan, in Kurdistan province, in 1980 (189). There was no further report of Tularemia until 2011, when tularemia seroprevalence was detected in butchers and other slaughterhouse workers in Sistan and Baluchistan province (189) and among children in Chaharmahal and Bakhtiari province in southwestern Iran (194). In another survey in 2011–2012, 14.4% of seropositivity was shown among high-risk groups in Kurdistan province (195).

Considering the fact that tularemia is reported in neighboring countries and in different serological studies in Iran, improving the surveillance of the infection seems necessary and awareness of physicians and healthcare workers of the natural life cycle of *F. tularensis* and its clinical manifestations is highly recommended.

#### Leptospirosis.

Leptospirosis is a zoonotic disease, caused by *Leptospira* spp., with a worldwide distribution. It has reemerged during the last decades in Iran (196, 197). The first isolation of *Leptospira* from men and cattle was performed in 1959 in this country (198). The infection was recorded in humans and animals in different regions of the country from 1959 through 1987 (199–202); the infection then re-emerged in Gilan, northern Iran, when 79 patients were confirmed for Leptospirosis in 1999 (203).

Today the seropositivity is found in humans and in a vast range of animals living in different parts of the country including Tehran, Azerbaijan, Khorasan, Isfahan, Chaharmahal and Bakhtiari and Bushehr provinces and Leptospirosis shows an endemic panel in Gilan and Khuzestan provinces in the north and southwest of Iran (204). The local Iranian serovars are *L. grippotyphosa, L. canicola, L. sejreohardjo, L. pomona* and *L. icterohaemorrhagiae* (205, 206).

In addition, Leptospirosis often remains unrecognized in most parts of the country, because of its unspecificity of sign and clinical symptoms. However, 14 to 53% of seropositivity is shown in different studies among the Iranian population (207–210). Most cases of the disease are reported in Mazandaran province, northern Iran (206).The main risk factors of the disease are working in paddy, keeping animals, contact with rodents and swimming in rivers (208, 209).

Different surveys of animals have revealed the Leptospirosis infection rate to be up to 43% in horses (211, 212), 37% in cattle (213–217), 37 % in dogs (218), 18.5% in sheep and goats (219, 220), as well as infections in cats and donkeys (212, 221, 222).

Mazandaran, Gilan and Khuzestan provinces are ideal areas for transmission of this infection in Iran as a result of high humidity, high population, and rural agricultural (mostly rice farming) and fishing economic conditions (223).

Instruction of preventive measures to rice farmers, animal keepers and persons living and/or finding themselves near rivers can decrease the infection transmission to humans in the endemic regions of the country.

#### Multidrug-Resistant Tuberculosis.

Tuberculosis (TB) has always caused great problems for health-care systems globally, significantly in un-deveopled and developing countries. On average and annually, approximately 3.6% of 8.6 million new cases of TB are thought to be multidrug-resistant tuberculosis (MDR-TB) in the world (224). MDR-TB is the form of *Mycobacterium tuberculosis* that is resistant to at least Isoniazid and Rifampin, the two most powerful first-line anti-TB drugs (225). Emergence and spread of MDR-TB has become a threat to TB control strategies during the last two decades (224).

In Iran, the first MDR-TB cases were reported in Tehran in 2000 in a hospital where patients with failure treatment were admitted. In this study, about 4% of cases with recently detected TB were resistant to the first line TB antibiotics (225). At the same time, 14% of previously treated cases were diagnosed as MDR-TB in East Azerbaijan, northwestern Iran (226).

After establishment of national TB control programs in 1996, MDR-TB was found in 5.1% of new and 33.7% of retreated TB cases in Iran (224). In a study from 2010 to 2012, performed in five provinces (Tehran, Sistan and Baluchistan, Kermanshah, Hormozgan and Isfahan), it was revealed that 5% of the Ural type isolates were MDR-TB (227). The highest prevalence of MDR-TB was reported in Sistan and Baluchistan province (11.5%) and Isfahan (6.5%) in 2005 (228–230). Afterwards, this rate decreased amongst new cases, but the increasing trend was seen in previously treated patients (178, 231–233). Yet, MDR-TB has remained as a major challenge in TB controlling programs (233).

The trend of TB incidence has considerably shown lower rates in Iran than in the neighboring countries during the last decade and such an incidence has declined from 36 per 100,000 to 24 per 100,000 cases in 1990 and 2013, respectively. Sistan and Baluchistan province has the highest rate of MDR-TB rates among all provinces (224).

In 2006, the existence and transmission of extensively drug-resistant tuberculosis (XDR-TB) among patients with MDR-TB were reported in Iran (234, 235).

Introduction of rapid tests for MDR-TB detection in addition to effective drugs is required for the treatment of these patients.

#### Nontuberculous mycobacteria.

Nontuberculous mycobacteria (NTM) infection is a public health problem in different parts of the world (236). NTM, also known as atypical mycobacteria, was recognized in the 19^th^ century (237). Continuation of TB and MDR-TB led to the identification of NTM in Iran and reports of NTM infection rapidly increased due to the growing epidemics of HIV infection and the significant improvements in laboratory diagnostic methods (238, 239).

In Iran, however, NTM infection was identified among 11.43% of all patients suspected to have MDRTB from 2002 to 2006 (240). *Mycobacteria fortuitum, M. kansasii, M. abscessus* and *M. avium* complex have been shown as the most frequent types of NTM in Iran (241). Since then, multiple species have been reported as emerging agents in the country; *M. parascrofulaceumin* (242), *M. lentiflavum* (243), *M. conceptionense* (244) and *M. novocastrense* (245), *M. monacense* (246), *M. setense* (247), *M. elephantisfrom* (248), *M. europaeum* (249), *M. chelonae* (250) and *M. iranicum* (251), *M. celeriflavum* (252) are recently isolated NTMs in Iran.

The misdiagnosis of NTM infection with MDR-TB may lead to wrong treatment of TB patients, so improvement of NTM laboratory detection methods is highly recommended in all parts of the country.

#### Glanders.

Glanders, caused by *Burkholderia mallei*, is a zoonotic bacterial disease that occurs primarily in horses, mules and donkeys. This highly contagious pathogen is transmitted to humans by direct contact with infected animals and entry is through skin abrasions, nasal and oral mucosal surfaces, or by inhalation (253, 254). The occupational transmission of glanders can occur among farmers, veterinarians and laboratory technicians (255).

The history of glanders in Iran goes back to about a century ago when the disease was reported from almost all parts of the country. In this regard, Iran has conducted a national campaign of the test and slaughter of infected equines by *B. mallei* since 1961 by using mallein test. The outbreak of disease was reported among horses and humans in Kurdistan in 1974. After that, the infection was not reported up to 1994 when it was confirmed among horses in the central part of the country (256). Afterward, some cases of glanders were reported when four African lions and one Siberian tiger died in Tehran zoo with clinical signs of the disease in 2012 (257).

Unfortunately this disease has either no vaccine or immunization method and the main controlling program is based on preventing any movement of infected equines and slaughtering of the confirmed animals.

Consequently, physicians must be aware of the clinical symptoms of glanders in case of visiting a patient with a history of equine contact in order to best diagnose the disease.

### Parasitic and fungal EIDs and RID

#### Fasciolosis.

Fasciolosis is a zoonotic disease caused by the liver flukes of the genus *Fasciola* (258). *Fasciola hepaticais* is present in Europe, Africa, Asia, the Americas while *Fasciola giganticais* has been detected in Africa and Asia (259). Most human cases of Fasciolosis in Asia have been reported from Iran (260).

Fasciolosis was reported as an emerging disease in Gilan Province, northern Iran, in 1995 and the number of cases increased up to 10,000 patients in 1999 (261–263). The disease is at endemic levels in Mazandaran province, neighboring Gilan, and Khuzestan province, in the southwest of the country (264–267); such a similarity in the pattern of the disease arises from similar climatic conditions and animal husbandry management in these provinces. The disease is also reported among humans and animals in other provinces such as Kurdistan, Zanjan, Tehran and Azerbaijan (259). The emergence of Fasciolosis, with renal failure, was reported in Kermanshah in 1998 (268). Later on, a new human outbreak was reported in Yasuj district in southwestern Iran (269).

*F. hepatica* and *F. gigantica* were isolated in different provinces of Iran; the most prevalent species, to date, has been *F. gigantica* (38.5–62%). Most liver condemnations due to a Fascioliasis infection are reported in slaughtered cattle (266–268, 270).

Aquatic snails of Lymnaeidae family are the intermediate hosts in the transmission of liver flukes (268). Ingesting contaminated, uncooked, fresh aquatic vegetables and water are the major sources of infection transmission among Iranian patients (271, 272).

Accurate diagnosis of infected animals and snails plays an important role in the control measures of Fasciolosis in livestock and humans in different parts of the country.

#### Drug Resistant Malaria.

Malaria is a major global health problem. The most severe forms of the disease and almost all of related deaths from Malaria are due to *Plasmodium falciparum* (273). It has had an endemic pattern in the southern and southeastern regions of Iran, including Hormozgan, southern Kerman and Sistan and Baluchistan provinces (274). Consequently, drug resistant malaria is considered as a challenge in malaria control and elimination programs in these endemic regions (275).

Studies that have assessed the response of *P. falciparum* to chloroquine in the endemic region from 1968 to 1976 revealed that all malaria isolates were sensitive to chloroquine (180, 276). Yet, chloroquine-resistant cases were found for the first time among 5.7% of the infected cases in 1983 in the Iran-Shahr district and Sistan and Baluchistan province (277). The rate of drug resistant malaria increased to almost 50% in south-eastern Iran in 1996 and 68%–84% during the period 1997–2001 in the south and southwest of the country (275, 278, 279). It is supposed that the resistant strains of *P. falciparum* were most probably originating from Southeastern Asia (280). In 1999, the rates of resistance to chloroquine, amodiaquine, sulfadoxine-pyrimethamine in the endemic regions were 33.4%, 15.2%, 17.9%, 2.2%, respectively (275). A sulfadoxine-pyrimethamine (SP) combination was introduced as the first-line drug after the development of resistance to chloroquine in the country (275).

During the period between 2000 and 2010, studies conducted in endemic provinces revealed a decreasing trend in Malaria while most of the cases were still chloroquine-resistant. Moreover, the tested samples have shown no sulfadoxine–pyrimethamine resistant in Iran (275, 281).

Although the reported cases of Malaria has decreased to less than 100 cases during the recent years, conducting continued routine drug resistance surveys are necessary in endemic regions to have updated information regarding the situation of Malaria resistant species in Iran.

#### Microsporidiosis.

Microsporidiosis is an opportunistic intestinal infection caused by a group of obligatory intracellular parasitic fungi (282). It is considered as an emerging infectious disease (283) which is most frequently reported among immunocompromised people (282). It often results in weight loss and wasting syndrome and in certain cases in developing countries, it leads to death (284).

Since 1985, several reports have shown intestinal microsporidiosis due to *Enterocytozoon bieneusi*, a microsporidian species, as a frequent cause of chronic diarrhea amongst Iranian HIV positive patients (285).

More recently, microsporidiosis has been reported among 2.5 to 30% of Iranian HIV/AIDS patients in different studies (284–286). Microsporidiosis is also reported among chronic psychiatric cases and respiratory complicated patients (287).

By improvement of microsporidiosis diagnostic methods through higher sensitive techniques, it is expected that more cases be reported in the country in future.

## CONCLUSION

With rapidly increasing movement of people, pathogens, and vectors across borders, EIDs and RIDs are regularly introduced as public health concerns. Improvement of the surveillance system is needed to predict that such diseases would emerge in a special situation or during particular duration.

As some of the EIDs or RIDs in Iran are vector borne diseases such as Crimean Congo Hemorrhagic Fever, West Nile Fever, Dengue Fever, Q fever, Plague and Tularemia, it is highly needed to improve the vector surveillance system in the country in order to have a better monitoring and early warning systems for these diseases. Vectors in high risk areas and endemic regions should be systematically surveyed for further interventions and vector control programs should be implemented if required.

Some other EIDs or RIDs in Iran are blood borne or sexually transmitted diseases such as HIV/AIDS, Hepatitis C, Occult Hepatitis B and Human T-cell leukemia; consequently, continues bio-behavioral surveys among high risk groups are essential to have a better view of their epidemiology and trend leading to better management and controlling. Moreover, one of the most successful preventive methods for these diseases is to increase the level of knowledge and awareness of different populations regarding preventive methods; hence, increasing training programs is highly recommended to more or less control these diseases in the country.

Other EIDs and RIDs in Iran are zoonotic diseases such as Leptospirosis, Glanders, Hantaviruses and Middle East respiratory syndrome. Controlling these aforementioned diseases necessitates a close collaboration of the Ministry of Health and the veterinary organization, since animals are the main reservoirs of theses disease. Presenting instructions about preventive measurements to occupations at high risk of these diseases plays an important role in decreasing the infection transmission to humans in endemic regions of the country. It is recommended to do more seroepidemiological surveys in animals in high risk areas in order to identify the true situation and risk factors for acquiring these zoonotic diseases.

For diseases such as Multidrug-Resistant Tuberculosis and Drug Resistant Malaria, continuing surveys on routine drug resistance is necessary in endemic regions. These emergencies bold the importance of appropriate use of antibiotics to reduce the probability of resistant pathogens in the country. Further efforts should be directed towards increasing the awareness of physicians in rapidly diagnosing, reporting and prescribing the correct drugs and antibiotics for treating these diseases. As Afghan refugees have an important role in importing and spreading of Multidrug-Resistant Tuberculosis and Drug Resistant Malaria in Iran, it is highly recommended to monitor the health status of Afghan immigrants when entering Iran, to reduce the spread of these diseases.

Developing specialized clinical laboratories in all parts of the country based on the reported EIDs and RIDs in the related regions is an important basis to diagnose the diseases in the shortest possible time. The development of an integrated response to multiple threats posed by climatic changes, vector-borne diseases, and emerging threats seems to be a realistic way forward. To achieve such integration, the Iranian Ministry of Health and other related organizations must increase investments in better data quality, methodologies, and tools to provide improved information services across key disciplinary areas (climate, environment, pathogens, people, vectors, livestock, etc.) with a primary focus on serving national decision-making needs.
